# Predicting Suicide Risk in Trauma Exposed Veterans: The Role of Health Promoting Behaviors

**DOI:** 10.1371/journal.pone.0167464

**Published:** 2016-12-21

**Authors:** Bryann B. DeBeer, Julie A. Kittel, Andrew Cook, Dena Davidson, Nathan A. Kimbrel, Eric C. Meyer, Suzy B. Gulliver, Sandra B. Morissette

**Affiliations:** 1 Department of Veterans Affairs, VISN 17 Center of Excellence for Research on Returning War Veterans, Waco, Texas, United States of America; 2 Texas A&M Health Science Center, Temple, Texas, United States of America; 3 Central Texas Veterans Health Care System, Temple, Texas, United States of America; 4 Durham Veterans Affairs Medical Center, Durham, North Carolina, United States of America; 5 VA Mid-Atlantic Mental Illness Research, Education, and Clinical Center, Durham, North Carolina, United States of America; 6 Duke University School of Medicine, Durham, North Carolina, United States of America; 7 Warriors Research Institute, Baylor, Scott & White Healthcare System, Waco, Texas, United States of America; Stellenbosch University, SOUTH AFRICA

## Abstract

**Introduction:**

Returning veterans of the wars in Iraq and Afghanistan experience high rates of post-traumatic stress disorder (PTSD) and suicidal behavior. Suicidal ideation is among the strongest risk factors for completed suicide. Some research suggests an association between PTSD and suicidal ideation, and that health-promoting behaviors—behaviors that sustain or increase well-being—play a role in this association. The current study examined whether health-promoting behaviors moderate the association between PTSD severity and suicidal ideation.

**Methods:**

Veterans of Operation Enduring Freedom/Operation Iraqi Freedom (OEF/OIF; N = 108) completed measures of PTSD symptoms, trauma exposure, suicidal ideation, and health-promoting behaviors.

**Results:**

Moderated regression was used to test the hypothesis. Results indicated that health promoting behaviors, *β* = -.06, p = .001, and PTSD symptoms, *β* = .36, p < .001, were significantly related to suicidal ideation. Consistent with our main hypothesis, the health promoting behaviors x PTSD interaction term was significantly associated with suicidal ideation, *β* = -.09, p = .001. The overall model accounted for 13% of the variance in suicidal ideation. Among individuals with high PTSD symptom severity, those who engaged in more health promoting behaviors reported less suicidal ideation than those who engaged in fewer health promoting behaviors.

**Conclusions:**

Health-promoting behaviors could be important for reducing suicidal ideation among veterans with high levels of PTSD symptoms. It is recommended that future research examine health promotion interventions as a means of reducing suicidal ideation.

## Introduction

In a recent survey, veterans identified suicide as the most formidable challenge they face [1]. Sadly, approximately 22 veterans die by suicide every day [2]. Contributing to this problem, one of the most common mental health diagnoses among veterans returning from the current war theatres is posttraumatic stress disorder (PTSD), which is a known risk factor for both suicidal ideation and behavior [3,4,5,6]. Notably, suicidal ideation is one of the strongest, and often proximal, predictors of suicide attempts and completions [7,8]. Further, major depressive disorder, which is highly comorbid with PTSD [9], independently increases risk for suicidal ideation and attempts [10]. Thus, identifying modifiable factors that could reduce the association between PTSD symptoms and suicidal ideation is a high research priority.

Health-promoting behaviors are one such factor that may influence the relationship between PTSD and suicidal ideation. Health-promoting behaviors include those that promote nutrition, physical activity, stress management, spiritual growth, health responsibility, and interpersonal relationships [11]. Nutrition and physical activity refer to choosing healthy foods and regular participation in exercise. Stress management consists of engaging in relaxation and strategies that reducing tension. Spiritual growth refers to the development of inner resources and working towards goals. Health responsibility involves attention to and accountability for one’s health. Finally, the interpersonal relations dimension focuses on closeness, intimacy, and communication with others. One organizational framework for understanding health related behaviors is the Health Promotion Model, in which health is defined as a positive dynamic state rather than simply the absence of disease [12]. In this model, cognitive-perceptual factors, such as importance and perceived control of health, and modifying factors, such as interpersonal relationships and health behavior, are important predictors of health [12]. Prochaska’s model of health behavior change posits that while health promoting behaviors are amenable to change, it is unlikely without intervention [13].

Several of the health-promoting behaviors, including physical activity, aspects of stress management, and interpersonal relationships, have been demonstrated to decrease suicidal ideation [14,15,16,17,18,19,20,21]. Those who engage in physical activity and/or participate in sports are at lower risk for suicidal ideation than those who do not [14,15]. Related to spiritual growth, individuals who feel they have a purpose in life reported lower suicidal ideation than those who did not [17]. In terms of social relationships, there is a significant body of literature supporting social support as a protective factor for suicidal ideation [18,21]. Zhang and colleagues [19] found that suicide attempters tended to have diets lower in dietary fiber and polyunsaturated fat compared to individuals who had no history of suicide attempts. Further, men and women who had prior suicide attempts were more likely to have insufficient intake of vegetables and fruits respectively compared to non-attempters [20]. To our knowledge, no study has examined a broad spectrum of health-promoting behaviors in relation to suicidal ideation, or in the context of PTSD.

Evidence supports a similar relationship between health-promoting behaviors and PTSD symptoms [22,23,24]. Adults who participated in an exercise intervention showed decreased levels of PTSD symptoms compared to baseline, and this reduction lasted after the intervention was completed [22]. Veterans with PTSD who felt they had purpose and meaning in life had better outcomes than those who did not [23]. Social support is associated with lower PTSD symptom severity in trauma-exposed individuals [24]. Disrupted sleep is a core symptom of PTSD [25,26], and research demonstrates that cognitive-behavioral treatments that reduce insomnia and nightmares can reduce other symptoms of PTSD[27,28]. However, there is a paucity of research examining the link between nutrition/health promoting behaviors, PTSD symptoms, and their association with suicidal ideation.

## Objectives & Hypotheses

The current study tested the hypothesis that health promoting behaviors moderate the association between PTSD symptom severity and suicidal ideation among Iraq/Afghanistan veterans, such that veterans who experienced higher PTSD severity and engaged in more frequent health promoting behaviors would have lower levels of suicidal ideation in comparison with veterans who experienced higher PTSD severity but engaged in less frequent health promoting behaviors. Thus, we hypothesized that health-promoting behaviors would buffer the association between PTSD symptom severity and suicidal ideation.

## Materials and Methods

### Participants

Veterans who served as part of Operations Enduring and Iraqi Freedom (OEF/OIF; N = 145) and who were enrolled for healthcare at the Central Texas Veterans Health Care System (CTVHCS) were recruited to participate in a study that assessed warzone stressors and post-deployment adjustment. Participants were recruited through direct mailings, flyers at CTVHCS and through presentations to Department of Veterans Affairs (VA) staff (e.g., primary care, mental health, and OEF/OIF program) and veterans’ service organizations. Participants were eligible if they were: (a) an OEF/OIF veteran; (b) able to provide informed consent; and (c) able to complete the full assessment battery. Exclusionary criteria included: (a) a diagnosis of bipolar or psychotic disorder; (b) recently began (i.e., had not reached stabilization) psychiatric medications or psychotherapy; or (c) suicidal or homicidal ideation, intent or plan that warranted crisis intervention. No participants were excluded based on needing crisis intervention, and individuals experiencing suicidal ideation (in the absence of imminent threat) were eligible. Fifteen participants were deemed ineligible (7 unable to complete baseline assessment, 6 diagnosis of bipolar disorder, and 2 with psychotic symptoms), resulting in a final sample of 130 eligible participants. The Columbia Suicide Severity Rating Scale was added later in the study, thus, 116 veterans were administered this measure. Further, data was missing on the CAPS for two participants and for 6 participants on the HPLP. Therefore, 108 veterans completed the relevant study measures.

### Procedures

All procedures were approved by the local Institutional Review Board. Telephone screens were conducted to determine initial eligibility. Veterans were then scheduled for a face-to-face assessment, at the outset of which written informed consent was obtained. Final eligibility was confirmed after completing study procedures, including a semi-structured diagnostic interview and self-report questionnaires.

Clinical interviewers in the study were all masters-level or above and underwent a rigorous training process on the Clinician Administered PTSD Scale (CAPS) and Columbia Suicide Severity Rating Scale (CSSRS). Raters were supervised by doctoral level clinicians. All raters were provided with a CAPS and CSSRS administration manual for the study, journal articles on the CAPS and CSSRS, and the CAPS and CSSRS instruments, and were then provided an in person training detailing the content of the measure and the administration of the measure. Following this training, raters observed a trained rater administer the CAPS for a minimum of two sessions or more until they felt comfortable administering the measure. Raters administered the measure with a trained rater present until they were able to administer the CAPS to criterion. The assessor was required to administer two CAPS at criterion before they were certified as an interviewer. After the assessor was certified and conducting interviews on their own, each interview was reviewed by a doctoral level psychologist with the rater present to ensure proper administration and accurate diagnosis.

### Measures

#### Eligibility

Sections of the Mini International Neuropsychiatric Interview (MINI) [29] were used to screen for bipolar disorder and psychotic disorders.

#### Demographic information

A demographic questionnaire created for the study assessed participant characteristics, including age, gender, race/ethnicity, relationship and cohabitation status, education, employment, income, and military service.

#### Trauma exposure

Trauma exposure was measured using the Full Combat Exposure Scale (FCES) [30] and the Trauma History Questionnaire (THQ) [31]. All participants were veterans who served in support of the conflicts in Iraq and/or Afghanistan. The vast majority of participants indicated they experienced potentially-traumatic events (PTEs) while deployed to a warzone (n = 105, 97.2%). The three participants who did not report exposure to potentially-traumatic combat events endorsed exposure to potentially-traumatic events while they were a civilian, as measured by the THQ. Thus, the sample was comprised of veterans who were exposed to PTEs.

#### Ptsd symptoms

The CAPS [32] is a semi-structured diagnostic interview. The CAPS assesses 17 symptoms of PTSD (as defined by the Diagnostic and Statistical Manual, Fourth Edition (DSM-IV[25]) and their intensity and frequency. The CAPS focused on the worst traumatic event that occurred during an OEF/OIF deployment. On the rare occasions that no such event could be identified, the CAPS was conducted based on general OEF/OIF warzone stress in order to obtain continuous PTSD symptom severity scores for all participants. Veterans were asked about their PTSD symptoms within the past 30 days, and to identify the worst month related to the event in which symptoms were most severe. The CAPS yields both a categorical PTSD diagnosis and a continuous symptom severity score. Internal consistency of the CAPS current symptoms in the current study was 0.98.

#### Suicidal ideation

The CSSRS [33] is a clinical interview designed to measure suicidal ideation and behavior (including attempts, aborted or interrupted attempts, and non-suicidal self-injury). The CSSRS assesses for severity of suicidal ideation (range 0–5) and attempts in the last 30 days, as well as lifetime history.

#### Health promoting behaviors

Health promoting behaviors were assessed using the Health Promoting Lifestyle Profile (HPLP) [11]. The HPLP is a 52-item measure that asks about several dimensions of current health promoting behaviors, including nutrition (e.g. “Choose a diet low in fat, saturated fat, and cholesterol”), spiritual growth (e.g., “Feel I am growing and changing in positive ways”), stress management (e.g., “Take some time for relaxation each day”), interpersonal relations (e.g., “Discuss my problems and concerns with people close to me”), physical activity (e.g., “Follow a planned exercise program”), and health responsibility (e.g., “Report any unusual signs or symptoms to a physician or other health professional”). Responses range from 1 (Never) to 4 (Routinely). Items are totaled and then averaged to calculate the total and subscale scores. Internal consistency of the full scale was 0.96. As there is a robust association between social support and suicidal ideation [34], additional analyses were conducted to ensure that the variance was not being carried by the interpersonal relations subscale of the HPLP-II. First, a total mean score for the HPLP-II was calculated, leaving out the interpersonal relations subscale. Then, the same data analytic strategy was applied using the newly calculated HPLP-II total mean score. Results indicated the same pattern as the prior analyses such that health promoting behaviors moderated the association between PTSD symptoms and current suicidal ideation.

#### Data analytic plan

Moderation analyses were conducted within *Mplus* [35]. Maximum likelihood estimation with robust standard errors (MLR) was used to estimate the parameters because this type of approach is more robust against moderate violations of assumptions.

## Results

### Participant Characteristics

The mean age of the final sample (*N* = 108) was 38.4 years (SD = 11.07). Participants were primarily male (86.0%), with 28.7% identifying as from Hispanic ethnicity. With respect to racial distribution, 64.8% identified themselves as Caucasian, 17.6% as African-American, 0.9% as Hawaiian/Pacific Islander, and 10.2% as “Other” (categories were not mutually exclusive). The mean education level was 14.28 years (SD = 2.62 years). The majority served in the Army (81.5%), while 4.6% served in the Air Force, 9.3% in the Marine Corps and 6.5% in the Navy (categories were not mutually exclusive). Most veterans served in active duty (95.3%), while 46.1% served in the reserves and 15.7% in the National Guard (categories were not mutually exclusive). Participants identified their ranks at discharge as: 28.7% E1–E4, 35.2% E5–E6, 18.5% E7–E9, 4.6% O1–O3, 7.4% O4–O9, 1.9% WO1-WO5; thus, the sample was comprised of 13.9% officers. Of the sample, 44.4% were receiving medication management from a doctor or psychiatrist for a psychological condition, 35.9% were receiving individual psychotherapy, and 18.5% were engaged in group psychotherapy.

Forty participants (37.0%) met current and 61 (56.5%) met lifetime (worst-month) diagnostic criteria for DSM-IV military-related PTSD. A total of 5 participants (4.6%) had attempted suicide at some point in their lifetime, which rose to 15.7% (n = 17) when considering any history of suicidal behavior (i.e., attempts, aborted or interrupted attempts, preparatory acts/behavior). Of the sample, 10.1% endorsed suicidal ideation in the last 30 days. This is consistent with previous studies of suicidal ideation in populations containing Veterans both with and without PTSD (14% [36]). The mean suicidal ideation severity score within the past month was .20 (*SD* = .72; range: 0 to 5). As is typical with such data, CSSRS data were positively skewed (4.56, SE = .23) and kurtotic (23.20, SE = .46). Thus, CSSRS scores were log transformed, which reduced both skewness (3.38, SE = .23) and kurtosis (11.29, SE = .46). Scores on the HPLP ranged from 1.35 to 3.69. Mean total and subscale scores are presented in Table 1. Over the previous 4 months leading up to their study participation, 2.8% (*n* = 3) participants indicated they received individual psychotherapy only, 12.0% (*n* = 13) received medication management for a psychological condition through a psychiatrist or medical doctor only, 16.7% (n = 18) received both individual therapy and medication management, .9% (n = 1) received both group therapy and medication management, 1.9% (n = 2) received both individual therapy and group therapy, and 15.7% (n = 17) received all three types of treatment.

**Table 1:** Mean and Standard Deviation Scores of HPLP and Subscales.

Scale	Mean	SD
HPLP Total Score	2.33	0.54
Physical Activity Subscale	2.20	0.73
Nutrition Subscale	2.20	0.61
Health Responsibility Subscale	2.18	0.57
Spiritual Growth Subscale	2.65	0.72
Stress Management Subscale	2.24	0.59
Interpersonal Relations Subscale	2.53	0.71

### Correlational Analyses

Correlations between PTSD, SI, and health-promoting behaviors are presented in Table 2. The strongest relationship for suicidal ideation was with PTSD symptom severity.

**Table 2:** Correlations Among Health Promoting Behaviors, PTSD, and Suicidal Ideation.

Measure	1	2	3	4	5	6	7	8	9
HPLP Total	--	--	--	--	--	--	--	--	--
HPLP Physical Activity	.781**	--	--	--	--	--	--	--	--
HPLP Nutrition	.765**	.594**	--	--	--	--	--	--	--
HPLP Health Responsibility	.802**	.625**	.587**	--	--	--	—	--	--
HPLP Spiritual Growth	.872**	.557**	.522**	.590**	--	--	--	--	--
HPLP Stress Management	.861**	.708**	.589**	.617**	.731**	--	--	--	--
HPLP Interpersonal Relations	.825**	.445**	.478**	.574**	.856**	.672**	--	--	--
CAPS	-.560**	-.311**	-.440**	-.274**	-.610**	-.515**	-.550**	--	--
SI	-.352**	-.220*	-.299**	-.233*	-.414**	-.234*	-.326**	.339**	--

*Note*: HPLP = Health Promoting Lifestyle Profile; CAPS = Clinician-Administered PTSD Scale; SI = Suicidal Ideation.

* Significant at the .05 level.

**Significant at the .01 level.

### Moderated Regression Analysis

**Fig 1:**
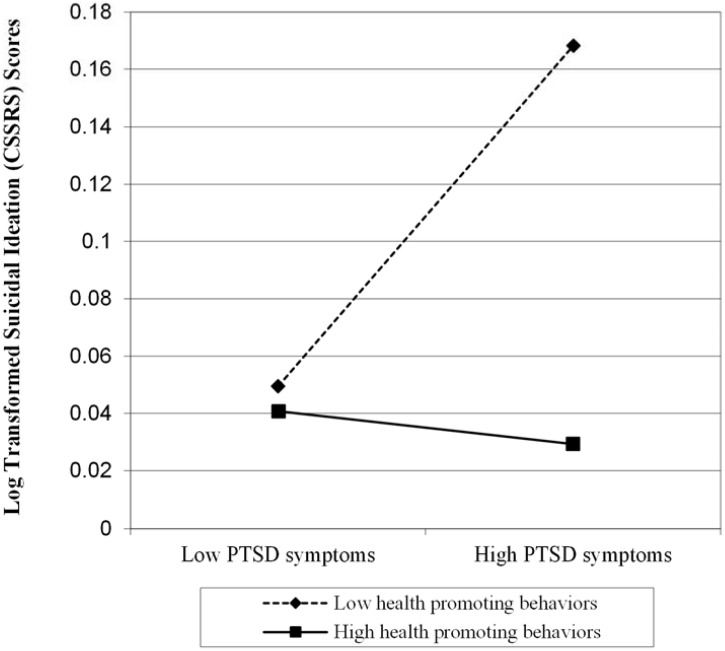
Plot of health promoting behaviors and PTSD symptoms interaction predicting suicidal ideation among Iraq/Afghanistan Veterans (N = 108). The dotted line reflects veterans who engage in health promoting behaviors infrequently, while the solid line represents veterans who frequently engage in health promoting behaviors. Log transformed suicidal ideation scores are on the y axis and PTSD symptoms are on the x axis.

Moderated regression was used to test the main hypothesis that health promoting behaviors moderate the effect of PTSD symptoms on suicidal ideation. The dependent variable was the log-transformed CSSRS past month suicidal ideation score. Gender and age were entered to control for possible differences in these characteristics across diagnosis and level of SI. Mean centered score for CAPS PTSD symptom severity and health promoting behaviors and a health promoting behaviors x PTSD interaction term (HBP x PTSD) were also entered into the model. Neither gender, *β* = -.09, *p* = .35, nor age, *β* = -.11, *p* = .27, were significantly related to suicidal ideation, and the overall model was not significant, *F*(2,105) = 1.03, *p* = .36. However, as expected, both health promoting behaviors, *β* = -.26, *p* = .02, and PTSD symptoms, *β* = .26, *p* = .02, were significantly related to suicidal ideation, and the overall model was significant *F*(4, 103) = 6.39, *p* < .001. In addition, consistent with our main hypothesis, the HBP x PTSD interaction term was significantly associated with suicidal ideation, *β* = -.24, *p* = .01, and the overall model in that step was significant, *F*(5, 102) = 6.89, *p* < .001. The final step of the model accounted for 6% of the variance above and beyond the prior steps. The overall model accounted for 22% of the variance in suicidal ideation. Consistent with the methods of Dawson et al., 2014, post-hoc probing of the interaction revealed that the association between PTSD symptoms and suicidal ideation was strongest when individuals endorse fewer health promoting behaviors, whereas the association between PTSD symptoms and suicidal ideation was weakest among individuals who did endorse engaging in high levels of health promoting behavior (Fig 1). Simple slope tests indicated that the association between PTSD symptoms and suicidal ideation was not significant when health promoting behaviors were higher (i.e., slope = -0.001, *t* = -1.052, *p* = 0.30). In contrast, when individuals endorsed engaging in fewer health promoting behaviors, the association between PTSD symptoms and suicidal ideation was significant (slope = .001, t = 2.025, p = .046).

## Discussion

The current study expands upon the present literature by explicating the role of health behaviors as a moderator of the association between PTSD symptoms and suicidal ideation. As found in prior studies, PTSD was associated with suicidal ideation. Next, we found an association between a broad spectrum measure of health-promoting behaviors and suicidal ideation. Results further indicated that when engagement in health promoting behaviors was high, PTSD had little impact on SI.

While the results are preliminary, our findings suggest that addressing health promoting behaviors from a broader perspective might be a useful area of intervention to reduce suicidal ideation in veterans with PTSD. Consistently, VA has implemented several wide-scale health behavior programs including a weight loss and nutrition consultation (MOVE) program. Our data suggest that the positive impacts of such health promotion programs could extend beyond physical health (e.g., reducing suicidal ideation). An area of future research would be to directly test whether health promoting interventions could reduce or prevent suicidal ideation, attempts, and suicide among those with PTSD. Moreover, health promoting behaviors could be expanded to include addictive behaviors (e.g., alcohol, tobacco use), which have profound effects on health and are commonly associated with PTSD [37,38] and suicide risk [39,40,41,42,43].

While our study has notable strengths, including the use of gold-standard, clinician administered assessment measures such as the CAPS and CSSRS, the results must be interpreted in the context of several limitations. Specifically, all participants were enrolled to receive care within the VA healthcare system. Therefore, it is unclear whether these findings extend to veterans who are not enrolled in VA healthcare or to non-veteran populations. In the broadest sense, VA healthcare enrollment could be considered a form of health-promoting behavior, although it is important to point out that not all veterans in the sample were treatment-seeking. Second, the design of the study was cross-sectional. Therefore, definitive conclusions regarding whether increasing health promoting behaviors reduces suicide risk cannot be made from the present study. Future research is needed to examine the impact of health promoting behaviors on suicidal ideation and behaviors in the context of a longitudinal design. Third, health promoting behavior was assessed through self-report. Thus, it is unclear if the health promoting behavior is the mechanism that explains this association or whether other associated behaviors such as attitudes or coping styles that are associated with a healthy lifestyle could better explain this finding. Future research is needed that includes more objective measures of health-promoting behavior (e.g., collateral reporters, behavioral measures, medical indicators of health), as well as mechanisms through which health promoting behaviors might operate (e.g., behavioral activation, physiological changes, increased self-efficacy, etc.) to decrease suicidal ideation. Further, only a small percentage of the sample endorsed suicidal ideation in the past 30 days (10.1%). Additionally, as the sample was primarily comprised of Army veterans, due to the geographical location of the study team, it is not representative of the broader OEF/OIF population. Finally, health promoting behaviors are only one among many potential factors that could moderate the association between PTSD symptoms and suicidal ideation. Future research is needed to examine complex relationships between health-promoting behaviors and other potential risk factors and moderators (e.g., coping, substance use, social support [34]). Unfortunately, in the current study, our sample size was not large enough to examine more specific aspects of health promoting behaviors.

Given the severity and scope of the problem of suicide among veterans, development of novel suicide prevention programs is a high priority. Whether these interventions should be comprehensive or targeted to specific populations is unknown, but an important area of future study. Therefore, we propose that the above-described type of interventions, such as nutritional consultations and the MOVE program, targeting health-promoting behaviors among veterans with PTSD who are at risk for suicide has potential value, and warrants further empirical investigation. Future research is needed to determine if the proposed interventions reduce suicide risk. Ideally, this research would employ a randomized controlled design in which half of participants are randomized to one of the health and wellness intervention mentioned previously, while the other half receives a control condition. In sum, this research provides a foundation for future inquiry into the effect of health promotion on suicide risk.
